# Exosomal lncRNA DLEU2 aggravates inflammatory injury and apoptosis in pediatric viral pneumonia via the miR-330-5p

**DOI:** 10.1186/s41065-026-00665-y

**Published:** 2026-03-25

**Authors:** Xinxing Su, Haobin Wang, Jianzhu He, Huagui Bai

**Affiliations:** 1 Department of Pediatrics, Affiliated Hospital of Yangzhou University, Yangzhou, 225000 China; 2 Pediatrics Department, The 960th Hospital of the PLA, Jinan City, Shandong Province 250031 China; 3 Pediatrics, The People’s Hospital Of Lincang, Lincang, 677000 China; 4 Department of Pediatrics, Chongqing Western Hospital, 301 Huafu Avenue North, Jiulongpo District, Chongqing, 400039 China

**Keywords:** Pediatric viral pneumonia, DLEU2, miR-330-5p, Biomarker

## Abstract

**Background:**

Viral pneumonia (VP) is a major cause of severe respiratory illness in children. lncRNAs are involved in disease progression by regulating immune regulation. This study investigates the clinical value and regulatory function of exosomal DLEU2 in pediatric VP.

**Materials:**

Include 247 pediatric VP cases, divided into 120 mild and 127 severe groups, and collected plasma exosomes samples. The abundance of DLEU2, miR-330-5p, and CEBPD were quantified using qRT-PCR. ROC curves and logistic regression were performed to determine the diagnostic and prognostic utility. The correlation was assessed between DLEU2 level and clinical indicators. A VP cell model was established by stimulating BEAS-2B with Poly(I: C). Cell transfection, ELISA, flow cytometry and dual-luciferase reporter assays were conducted to detect cytokines, apoptosis level and target gene regulation.

**Results:**

Exosomal DLEU2 was elevated in severe VP and showed good diagnostic performance in distinguishing severe from mild disease. High DLEU2 was independently associated with increased disease severity and correlated with adverse status, including tachypnea, respiratory retractions, reduced SpO_2_, and elevated inflammatory markers. Poly(I: C) stimulation upregulated DLEU2 and promoted cytokine IL-6 and IL-8 secretion and apoptosis in BEAS-2B. Silencing DLEU2 markedly attenuated these effects. DLEU2 functioned as a ceRNA by sponging miR-330-5p, thereby relieving its inhibitory effect on CEBPD. Restoration of miR-330-5p reversed DLEU2-mediated inflammatory and apoptotic responses.

**Conclusion:**

DLEU2 is upregulated in pediatric VP and is associated with disease severity. Functional assays suggest that DLEU2 may aggravate inflammatory injury in bronchial epithelial cells via miR-330-5p.

**Supplementary Information:**

The online version contains supplementary material available at 10.1186/s41065-026-00665-y.

## Introduction

Viral pneumonia (VP) represents a major subtype of community-acquired pneumonia in children. VP is a leading cause of respiratory infection-related morbidity, particularly among children under 5 years [1]. It is primarily characterized by inflammatory injury to the lung parenchyma following infection with diverse respiratory viral pathogens, often arising from the downward spread of upper respiratory tract infections [2]. The incidence of pediatric VP has increased markedly, driven by the diversification of viral strains and the influence of environmental factors [3]. Clinically, the disease is notable for its abrupt onset and rapid progression, with a subset of patients developing severe or critical illness [4]. Without timely intervention, critically ill children may rapidly deteriorate into respiratory failure or acute respiratory distress syndrome, posing a substantial threat to life [5]. Moreover, VP may result from single or multiple viral infections and presents with heterogeneous and often nonspecific early symptoms in children [6], which complicates early diagnosis, disease stratification, and clinical management.

LncRNA lack protein-coding potential but possess complex secondary structures enabling diverse regulatory functions. LncRNA have emerged as critical modulators of gene expression through their involvement in RNA interaction networks [7]. Accumulating studies have demonstrated that lncRNA play pivotal roles in inflammatory lung diseases and infectious pneumonia [7]. For instance, NFYC-AS1 modulates inflammatory responses by targeting miR-1323 in Mycoplasma pneumoniae pneumonia [8]. In the context of viral infection, elevated SNHG15 expression has been shown to promote SARS-CoV-2 propagation through the ACE2 pathway [9].

DLEU2 is implicated in the regulation of apoptosis and inflammatory signaling and has been associated with multiple pathological processes [10]. Notably, DLEU2 expression is significantly upregulated in critically ill patients with SARS-CoV-2 infection and has been identified as an immune-related aberrant lncRNA [11]. DLEU2 participates in chronic viral hepatitis and may serve as an inflammation-associated diagnostic biomarker, highlighting its relevance in virus-induced inflammatory responses [12]. Increased DLEU2 expression has been reported in idiopathic pulmonary fibrosis [13]. Given that viral pneumonia is characterized by excessive inflammatory activation and lung injury, these findings collectively suggest that DLEU2 may play a critical role in the progression of VP. Consistent with these observations, our preliminary data indicate that DLEU2 expression is markedly elevated in children with severe VP, implicating DLEU2 as a potential contributor to disease severity. DLEU2 can function as a ceRNA by binding specific miRNAs and thereby modulating downstream gene expression [14, 15]. Bioinformatic analysis using ENCORI predicts miR-330-5p as a potential target of DLEU2. miR-330-5p has been reported to be downregulated in SARS virus–infected tissues in animal models [16] and in hosts during parasitic infections, where it influences the expression of proinflammatory factors [17]. These findings collectively suggest that DLEU2 may regulate inflammatory responses in VP through modulation of miR-330-5p.

The present study aimed to investigate the clinical significance of DLEU2 in pediatric VP and to elucidate its underlying molecular mechanism. Plasma exosomal DLEU2 expression was detected in children with VP to evaluate its diagnostic value and association with clinical characteristics. Poly (I: C)-induced bronchial epithelial cell injury model was established to explore the functional effects of the DLEU2/miR-330-5p axis on inflammatory cytokine production and apoptosis. Downstream target genes were predicted and experimentally validated to clarify the regulatory network involved. This study sheds new light on the role of DLEU2 in pediatric VP and identifies a potential molecular axis that may contribute to disease progression and severity.

## Materials and methods

### Study population

Children diagnosed with VP and admitted to Chongqing Western Hospital between January and December 2024 were consecutively enrolled in this study. According to disease severity, patients were stratified into a severe (*n* = 127) and mild VP group (*n* = 120). Disease severity was determined in accordance with the Diagnosis and Treatment Guidelines for Community-Acquired Pneumonia in Children (2024).

Severe VP accompanied by at least one of the following criteria: (1) poor general condition; (2) signs of dehydration or refusal to feed; (3) altered mental status; (4) markedly increased respiratory rate, defined as ≥ 70 breaths/min (infants and toddlers) or ≥ 50 breaths/min (older children); (5) cyanosis; (6) dyspnea; (7) peripheral oxygen saturation (SpO₂) ≤ 0.92 at sea level; (8) extensive pulmonary infiltrates involving multiple lobes or ≥ 2/3 of a single lobe on imaging; (9) pleural effusion; or (10) presence of extrapulmonary complications.

Exclusion criteria: (1) age ≤ 28 days or ≥ 6 years; (2) primary or secondary immunodeficiency, genetic or metabolic disorders, or connective tissue diseases; (3) congenital heart disease; (4) chronic pulmonary conditions such as bronchiectasis, lung abscess, pulmonary bullae, or bronchopulmonary dysplasia; (5) congenital airway malformations; hematological disorders; tracheal foreign body aspiration; (6) prior history of recurrent wheezing or asthma; (7) and evidence of concurrent fungal infection, Mycobacterium tuberculosis, Mycoplasma pneumoniae, or Chlamydia infection during the current illness.

This study was approved by the Ethics Committee of Chongqing Western Hospital. Written informed consent was obtained from each subject prior to enrollment.

### Pathogen detection

Throat swab specimens were collected from all enrolled patients within 24 h of admission. Immediately after collection, samples were placed into sterile centrifuge tubes containing 2.5 mL of viral transport medium, stored at 4 °C during transportation to the laboratory to analyze, and subsequently preserved at − 80 °C backup. Polymerase chain reaction (PCR) assays were performed to identify common respiratory viral pathogens. Detailed pathogen detection results are provided in Table S1 (Supplementary Appendix).

### Sample collection

Peripheral blood samples were collected from patients within 24 h of admission using EDTA anticoagulant tubes. All samples were processed within 2 h after collection to minimize RNA degradation. Blood specimens were centrifuged at 3000 g for 15 min at low-temperature. The plasma supernatant was transferred to RNase-free tubes and centrifuged again at 12,000 g for 20 min at 4 ℃ to remove residual platelets, cell debris, and apoptotic bodies. The clarified plasma was either directly processed for exosome isolation or stored at -80 °C until further processing. Plasma exosomes were isolated using specialized exosome Kit (System Biosciences, USA) and used for downstream RNA extraction.

### Cell culture and VP cell model establishment

BEAS-2B cells were obtained from ATCC, (USA). Cells were cultured in DMEM (Gibco, USA) containing 10% FBS (Gibco) at 37 °C with 5% CO2. To establish VP model, BEAS-2B were inoculated into 6-well plates at a density of 2⋅10^5^ cells per well and allowed to adhere overnight. Cells were then stimulated with polyinosinic-polycytidylic acid [Poly(I: C); InvivoGen, USA] at 10 µg/mL to mimic viral-induced inflammatory injury.

### Cell transfection

Small interfering RNA targeting DLEU2 (si-DLEU2), miR-330-5p mimic, miR-330-5p inhibitor, and their corresponding negative controls were synthesized by GenePharma (Shanghai, China). 2⋅10^5^/well BEAS-2B cells were cultured in 6-well plates and transfected at approximately 70% confluence using Lipofectamine 3000 (Invitrogen, USA). The final concentration of siRNA was 50 nM, while miRNA mimics and inhibitors were transfected at 100 nM. Cells were harvested 24–48 h post-transfection for subsequent experiments.

### qRT-PCR detection

Total RNA, including lncRNAs and miRNAs, was extracted from plasma-derived exosomes using the miRNeasy Micro Kit (Qiagen, Germany). Total RNA was extracted from cell samples using TRIzol reagent (Invitrogen). RNA yield was assessed using a NanoDrop spectrophotometer (Thermo Fisher Scientific, USA). For DLEU2 detection, reverse transcription was performed using a PrimeScript RT Reagent Kit (Takara, Japan). For miR-330-5p analysis, cDNA synthesis was conducted using a miRNA-specific reverse transcription kit (Takara). qRT-PCR was carried out using SYBR Green PCR Master Mix (Takara) on an ABI 7500 System (Applied Biosystems, USA). PCR amplification was carried out in a 20 µL reaction volume following the manufacturer’s protocol. The amplification conditions were as follows: initial denaturation at 95 °C for 30 s, followed by 40 cycles of 95 °C for 5 s and 60 °C for 34 s. A melting curve analysis was performed to confirm specificity. The primer sequences were as follows (5′-3′): DLEU2 F: TCTGACCTGGTTACTCAGTCA, R: GCTGTCCTGGAACTCACTCT; miR-330-5p F: GCGTCTCTGGGCCTGTGTC, R: AGTGCAGGGTCCGAGGTATT; GAPDH F: TGTGGGCATCAATGGATTTGG, R: ACACCATGTATTCCGGGTCAAT; U6 F: CTCGCTTCGGCAGCACA, R: AACGCTTCACGAATTTGCGT.DLEU2. GAPDH and U6 were used as an internal reference. Relative expression levels were calculated using the 2^⁻ΔΔCt^ method.

### Cell apoptosis assay

Cell apoptosis was evaluated using an Annexin V-FITC/propidium iodide (PI) apoptosis detection kit (BD Biosciences, USA). BEAS-2B (1⋅10^5^ cells per sample) were treated with Poly(I: C) for 24 h before harvesting. Cells were washed with cold phosphate-buffered saline, resuspended in binding buffer, and stained with Annexin V-FITC and PI. Apoptotic cells were quantified using a BD FACSCanto II flow cytometer (BD Biosciences, USA). Early and late apoptotic cells were distinguished based on standard gating of Annexin V-FITC-positive/PI-negative and Annexin V-FITC-positive/PI-positive populations, respectively.

### ELISA

The concentrations of IL-6 and IL-8 in cell culture supernatants were measured using commercially available ELISA kits (R&D Systems, USA; IL-6: Catalog # D6050; IL-8: Catalog # D8000C) according to the manufacturer’s instructions. Absorbance was measured using a microplate reader. To ensure comparability between samples, cytokine levels were normalized to total cellular protein concentration, which was determined using a BCA Protein Assay Kit (Thermo Fisher Scientific; Cat. No. 23225).

### Dual-luciferase reporter assay

The candidate RNA binding sites (DLEU2 and miR-330-5p, miR-330-5p and CEBPD) were predicted using the ENCORI. Wild-type (WT) and mutant (MUT) fragments of DLEU2 or 3′UTR of CEBPD containing the putative miR-330-5p binding sites were cloned into the pmirGLO luciferase reporter vector (Promega, USA). BEAS-2B were transferred to 24-well plates at a density of 1⋅10^5^ cells per well and co-transfected with 500 ng of WT or MUT luciferase reporter plasmids and 100 nM miR-330-5p mimic or negative control using Lipofectamine 3000. After 48 h, luciferase activity was measured using a Dual-Luciferase Reporter Assay System (Promega).

### Statistical analysis

All statistical analyses were performed using R 4.4.0 and GraphPad Prism 9.0. Statistical analyses were performed using R software. Data visualization and figure rendering were conducted using GraphPad Prism. Data distribution was assessed for normality using the Shapiro-Wilk test, and homogeneity of variance was evaluated using Levene’s test. Data are presented as mean ± SD. Differences between two groups were analyzed using Student’s t-test. Comparisons among multiple groups were performed using one-way ANOVA, followed by Bonferroni’s post hoc test to correct for multiple comparisons. Categorical variables were analyzed using the chi-square test or Fisher’s exact test. Receiver operating characteristic (ROC) curves were generated to evaluate the diagnostic performance of DLEU2 (internal validation using 5-fold cross-validation). Pearson correlation and logistic regression analysis were conducted to evaluate the association performance and risk factors. The power analysis was conducted using a medium effect size (d = 0.5), a significance level of 0.05, and a desired power of 0.85 (two-tailed). The clinical sample size meets the requirements of the power analysis. P value < 0.05 was statistically significant.

## Results

### Clinical characteristics of pediatric VP

No significant differences were observed between the two groups in age or sex distribution (*P* > 0.05, Table 1). All children with severe VP exhibited tachypnea and respiratory retractions. Children with severe pneumonia exhibited higher incidences of crackles (*P* = 0.010) and reduced peripheral oxygen saturation (SpO_2_) (*P* < 0.001, Table 1). CRP and PCT were elevated in the severe population (*P* < 0.001, Table 1). Serum IL-6 levels were substantially increased in severe cases (*P* < 0.001), whereas IL-8 levels showed no significant difference between groups (Table 1).

**Table 1:** Baseline clinical characteristics of pediatric patients with mild and severe viral pneumonia

Parameters	Mild Pneumonia (*n* = 120)	Severe Pneumonia (*n* = 127)	*P*-value
Age, years	3.01 ± 1.53	2.97 ± 1.46	0.833
Sex, male, n	68	63	0.352
Clinical manifestations			
Fever, n	95	111	0.117
Cough, n	117	123	1
Tachypnea, n	-	104	-
Wheezing, n	48	61	0.253
Crackles, n	42	85	0.010
Respiratory retractions, n	-	86	-
SpO_2_, %	93.30 ± 0.87	89.88 ± 2.20	< 0.001
Inflammation marker			
WBC, 10^9^/L	8.59 ± 1.63	8.96 ± 2.10	0.124
CRP, mg/L	14.01 ± 6.34	26.64 ± 10.36	< 0.001
PCT, ng/mL	0.29 ± 0.08	0.49 ± 0.17	< 0.001
Cytokine			
IL-6	13.37 ± 5.73	43.41 ± 26.74	< 0.001
TNF-α	8.07 ± 0.68	8.18 ± 0.61	0.183

*Abbreviations:* *CRP* C-reactive Protein, *IL-6* Interleukin 6, *PCT* Procalcitonin, *SpO2 *Peripheral oxygen saturation, *TNF-α* Tumor Necrosis Factor alpha, *WBC* White blood cell

Respiratory virus composition analysis revealed distinct pathogen distributions between mild and severe pneumonia (Table S1). Severe cases exhibited a higher proportion of adenovirus (*P* = 0.035) and influenza A/B infections (*P* = 0.002), as well as a notably increased rate of mixed viral infections (*P* = 0.005, Table S1).

### DLEU2 is upregulated and exhibits diagnostic value in severe VP

**Fig. 1:**
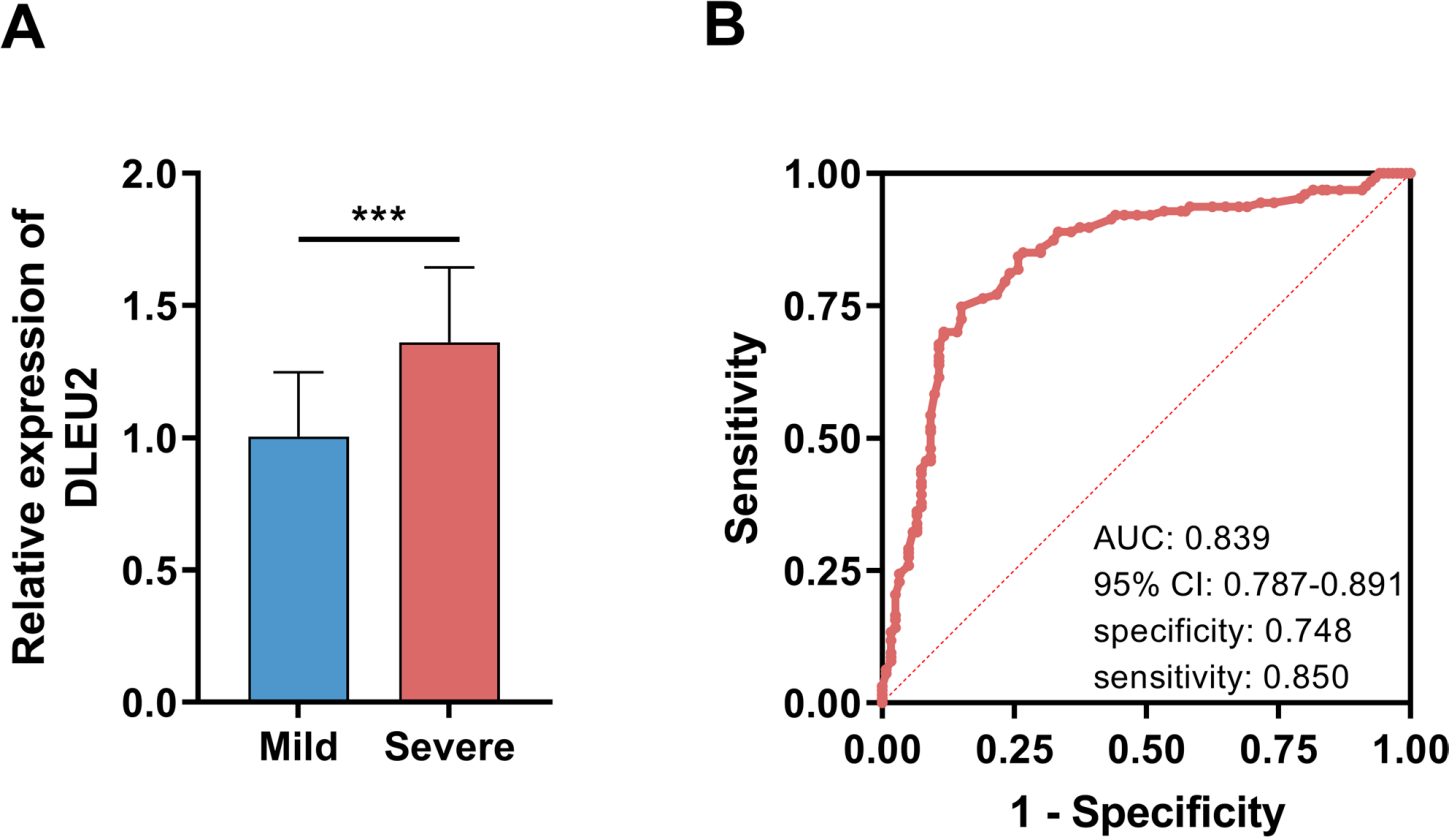
Plasma exosomal DLEU2 expression and diagnostic value in pediatric viral pneumonia (VP). **A** Relative expression of plasma exosomal DLEU2 in mild (*n* = 120) and severe pneumonia (*n* = 127) patients. **B** Receiver operating characteristic (ROC) curve analysis evaluating the diagnostic performance of DLEU2 for severe VP. Data are presented as mean ± SD. Comparisons between two groups were performed using Student’s t-test. *** *P* < 0.001

Plasma exosomal DLEU2 expression was higher in children with severe VP relative to those with mild disease (Fig. 1A, *P* < 0.05). DLEU2 possessed good diagnostic performance for distinguishing severe pneumonia (Fig. 1B). Internal validation supports the stability of the reported diagnostic performance (Figure S1).

### Association between DLEU2 and clinical indicators of disease severity

After adjustment for potential confounders, elevated DLEU2 remained significantly associated with disease severity (OR = 5.96, 95% CI: 3.06–12.28, *P* < 0.001), together with SpO2 (OR = 0.38, 95% CI: 0.19–0.76, *P* = 0.006), CRP (OR = 4.58, 95% CI: 2.32–9.42, *P* < 0.001), and IL-6 (OR = 4.54, 95% CI: 2.17–9.89, *P* < 0.001) (Table 2).

**Table 2:** Univariate and multivariate logistic regression analysis of risk factors for severe viral pneumonia

Parameters	Univariate			Multiple	
	OR value (95% CI)	*P*-value		OR value (95% CI)	*P*-value
Age	0.70 (0.38, 1.27)	0.249			
Sex	0.75 (0.45, 1.24)	0.266			
Fever	1.82 (0.92, 3.68)	0.084			
Cough	0.78 (0.15, 3.64)	0.758			
Wheezing	1.38 (0.83, 2.30)	0.204			
Crackles	1.88 (1.13, 3.16)	0.015		1.86 (0.96, 3.68)	0.067
SpO2	0.26 (0.15, 0.44)	< 0.001		0.38 (0.19, 0.76)	0.006
WBC	1.54 (0.93, 2.56)	0.088			
CRP	4.19 (2.48, 7.19)	< 0.001		4.58 (2.32, 9.42)	< 0.001
PCT	3.78 (2.23, 6.41)	< 0.001		1.44 (0.67, 3.21)	0.325
IL-6	5.69 (3.32, 9.97)	< 0.001		4.54 (2.17, 9.89)	< 0.001
TNF-α	1.23 (0.74, 2.03)	0.409			
DLEU2	6.47 (3.75, 11.43)	< 0.001		5.96 (3.06, 12.28)	< 0.001
miR-330-5p	0.43 (0.25, 0.72)	0.001		0.48 (0.24, 0.94)	0.034

*Abbreviations:* *CRP* C-reactive Protein, *IL-6* Interleukin 6, *PCT* Procalcitonin, *SpO2* Peripheral oxygen saturation, *TNF-α* Tumor Necrosis Factor alpha, *WBC* White blood cell

Patients were stratified into high- and low-DLEU2 expression groups based on the median DLEU2 level. High DLEU2 expression was associated with tachypnea (*P* = 0.035), respiratory retractions (*P* = 0.048), lower SpO₂ levels (*P* = 0.043, and elevated CRP (*P* = 0.022) and IL-6 concentrations (*P* = 0.007) (Table 3). No significant differences in age, sex, WBC count, or TNF-α (*P* > 0.05, Table 3).

**Table 3:** Association between plasma exosomal DLEU2 expression and clinical indicators in severe pneumonia

Parameters	Low expression (*n* = 66)	High expression (*n* = 61)	*P*-value
Age, years	2.92 ± 1.51	3.03 ± 1.42	0.677
Sex, male, n	29	34	0.249
Clinical manifestations			
Fever, n	56	55	0.525
Cough, n	64	59	0.429
Tachypnea, n	49	55	0.035
Wheezing, n	30	31	0.669
Crackles, n	31	33	0.531
Respiratory retractions, n	39	47	0.048
SpO_2_, %	90.26 ± 2.03	89.47 ± 2.31	0.043
Inflammation marker			
WBC, 10^9^/L	8.88 ± 1.93	9.04 ± 2.29	0.663
CRP, mg/L	24.63 ± 10.45	28.81 ± 9.90	0.022
PCT, ng/mL	0.47 ± 0.17	0.51 ± 0.16	0.180
Cytokine			
IL-6	37.38 ± 24.92	49.93 ± 27.34	0.007
TNF-α	8.10 ± 0.64	8.27 ± 0.58	0.110

*Abbreviations:* *CRP* C-reactive Protein, *IL-6* Interleukin 6, *PCT* Procalcitonin, *SpO2* Peripheral oxygen saturation, *TNF-α* Tumor Necrosis Factor alpha, *WBC* White blood cell

### Poly(I: C) induces DLEU2 expression, inflammatory cytokine release, and apoptosis in BEAS-2B cells

**Fig. 2:**
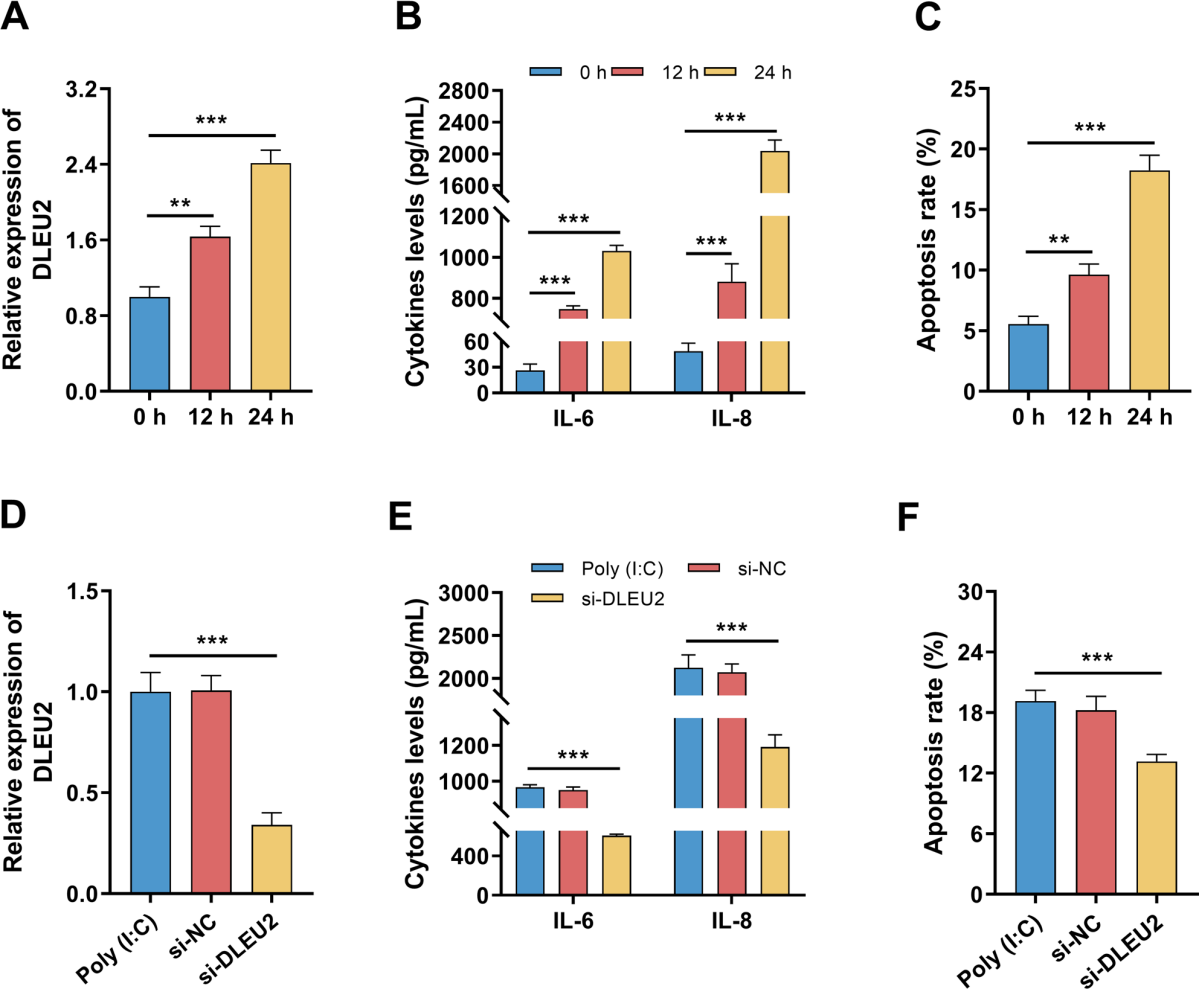
Effects of Poly(I: C) and DLEU2 knockdown on inflammation and apoptosis in BEAS-2B cells (*n* = 3). **A** Time-dependent DLEU2 expression following Poly(I: C) stimulation. **B** IL-6 and IL-8 secretion levels. **C** Apoptosis rates after Poly(I: C) treatment. **D-F** Effects of DLEU2 silencing on DLEU2 expression **D**, cytokine production **E** and apoptosis **F**. Data are presented as mean ± SD. Comparisons among multiple groups were performed using one-way ANOVA followed by Bonferroni’s post hoc test. ** *P* < 0.01, *** *P* < 0.001

DLEU2 expression was significantly increased in a time-dependent manner following Poly(I: C) stimulation (Fig. 2A, *P* < 0.05). The secretion levels of IL-6 and IL-8 were markedly elevated (Fig. 2B, *P* < 0.05), accompanied by a significant increase in apoptosis rates (Fig. 2C, *P* < 0.05). Silencing DLEU2 effectively reduced Poly(I: C)-induced DLEU2 expression (Fig. 2D, *P* < 0.05), attenuated IL-6 and IL-8 secretion (Fig. 2E, *P* < 0.05), and significantly decreased apoptosis in BEAS-2B cells (Fig. 2F, *P* < 0.05).

### DLEU2 functions as a ceRNA for miR-330-5p

miR-330-5p expression was downregulated in severe pneumonia patients and Poly(I: C)-treated BEAS-2B cells (Fig. 4A-B, *P* < 0.05). Low miR-330-5p (OR = 0.48, 95% CI: 0.24–0.94, *P* = 0.034) was an independent predictor for disease advancement (Table 2). The negative correlation was observed between DLEU2 and miR-330-5p expression (Fig. 4C, *P* < 0.001). Bioinformatic analysis predicted a direct binding site between DLEU2 and miR-330-5p (Fig. 4D). miR-330-5p mimic reduced luciferase activity of the DLEU2 WT reporter but not the MUT construct (Fig. 4E, *P* < 0.05). miR-330-5p overexpression partially reversed the pro-inflammatory and pro-apoptotic effects induced by DLEU2, whereas miR-330-5p inhibition abrogated the protective effects of DLEU2 knockdown in BEAS-2B (Fig. 4F-H, *P* < 0.05).

**Fig. 3:**
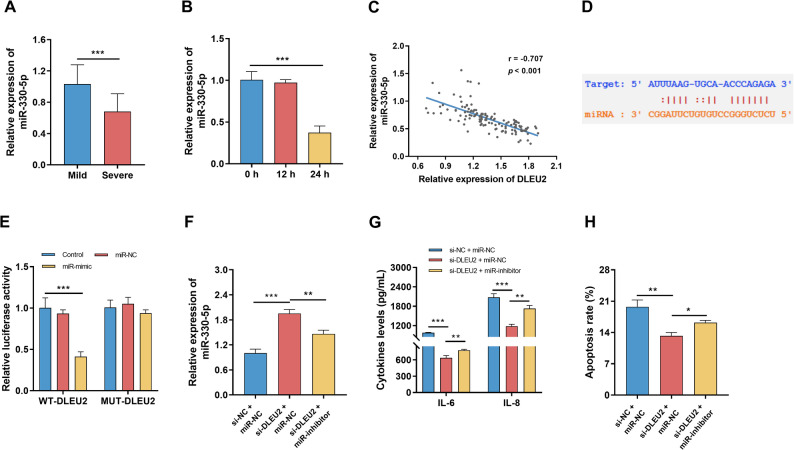
DLEU2 functions as a sponge for miR-330-5p. **A** miR-330-5p expression in mild (*n* = 120) and severe pneumonia (*n* = 127) patients. **B** miR-330-5p expression in Poly(I: C)-treated BEAS-2B cells (*n* = 3). **C** Correlation between DLEU2 and miR-330-5p expression (*n* = 127). **D** Predicted binding sites between DLEU2 and miR-330-5p. **E** Dual-luciferase reporter assay validating DLEU2–miR-330-5p interaction (*n* = 3). **F-H** Functional rescue experiments assessing miR-330-5p expression **F**, inflammation **G** and apoptosis **H** (*n* = 3). Data are presented as mean ± SD. Student’s t-test was used for two-group comparisons, and one-way ANOVA followed by Bonferroni’s post hoc test was used for multiple group comparisons. * *P* < 0.05, ** *P* < 0.01, *** *P* < 0.001

### miR-330-5p directly targets CEBPD

**Fig. 4:**
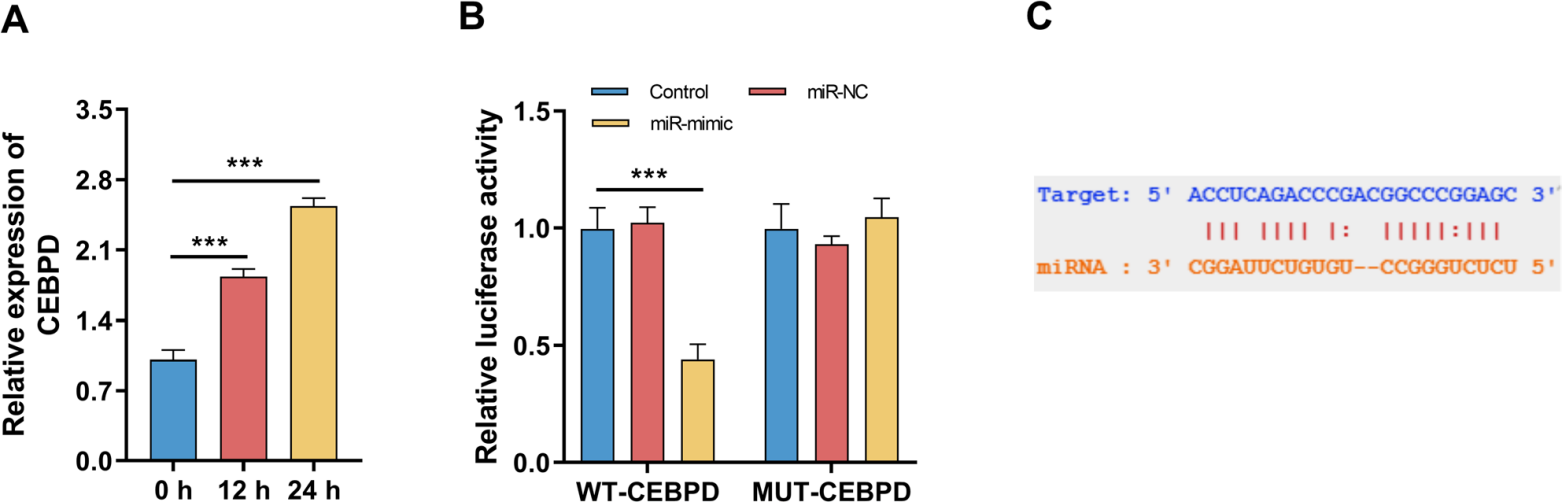
miR-330-5p directly targets CEBPD (*n* = 3). **A** CEBPD expression following Poly(I: C) stimulation. **B** Dual-luciferase reporter assays confirming the binding between miR-330-5p and CEBPD 3′UTR. **C** Predicted binding sites between CEBPD and miR-330-5p. Data are presented as mean ± SD. Comparisons among multiple groups were performed using one-way ANOVA followed by Bonferroni’s post hoc test. *** *P* < 0.001

CEBPD expression was significantly enhanced in Poly(I: C)-exposed BEAS-2B cells (Fig. 4A, *P* < 0.05). miR-330-5p directly bound to the 3′UTR of CEBPD, leading to reduced luciferase activity in the WT but not MUT groups (Fig. 4B-C, *P* < 0.05).

## Discussion

Immune systems of children are not yet fully mature, making them more susceptible to various viruses [4]. Children aged ≤ 6 years were included in this study because viral pneumonia is most prevalent and often more severe in this age group due to immature immune function and increased susceptibility to respiratory infections. Focusing on this population enhances the clinical relevance and homogeneity of the cohort. VP accounts for half or more of community-acquired pneumonia in children (especially those under 2 years old), and infections easily progress to severe pneumonia [2]. This study selected children aged 5 years and younger as subjects. Fever and cough are typical clinical symptoms following VP infection [18], with over 80% of patients in this study exhibiting these symptoms. Tachypnea and respiratory retractions are recognized core physical signs for screening severe pneumonia in children [19], serving as reference points for distinguishing severe from mild cases in this study. Severe patients exhibited a higher prevalence of crackles, potentially linked to higher rates of influenza and adenovirus infections in severe cases-viruses more likely to produce crackles on auscultation [20]. Additionally, severe VP causes more extensive alveolar and pulmonary interstitial exudation, contributing to crackle development [21]. SpO₂ directly reflects the degree of impaired pulmonary gas exchange [22]. A decline in SpO₂ in severe VP is a clear indicator of worsening disease. The correlation between DLEU2 expression levels and SpO₂, tachypnea, and respiratory retractions suggests DLUE2 may serve as a potential biomarker for assessing VP severity and directly or indirectly participate in the pathophysiological processes of severe pneumonia. Tachypnea and respiratory retractions often represent early symptoms of VP [19], suggesting DLUE2 detection may aid in identifying children who appear stable but are at high risk of progressing to severe disease. ROC analysis results validate this hypothesis, demonstrating DLUE2 exhibits good diagnostic performance in distinguishing severe from mild cases. After adjusting for clinical and laboratory indicators, DLUE2 remains an independent risk factor for severe VP. DLEU2 serves not only as a severity biomarker but also as a prospective tool for early risk stratification in pediatric patients.

PCT, CRP, and IL-6 serve as biomarkers for identifying pathogens, inflammatory status, and disease severity [23]. Most viruses do not directly activate PCT. Cytokine storms stimulate non-thyroid tissues to express PCT in severe viral infections, resulting in mild elevations in advanced patients [24, 25]. CRP and IL-6 expression is upregulated in this study. CRP responds rapidly to tissue injury and inflammatory reactions. Compared to bacterial infections, CRP shows a mild elevation in viral infections, making it useful for initial screening of inflammation severity and infection type [26]. IL-6 reflects the immune activation state and is suitable for assessing inflammation intensity [27]. High DLUE2 expression correlates with the upregulation of these indicators, suggesting this molecule is likely a key regulatory factor driving excessive inflammatory responses and pathological damage in severe pneumonia.

A Poly(I: C)-induced VP model was established in BEAS-2B cells to investigate the function of DLEU2. VP primarily affects lower respiratory epithelial cells. BEAS-2B cells, derived from human bronchi, offer higher reproducibility than primary cells [28]. Poly(I: C), a synthetic double-stranded RNA analog, is widely used to mimic virus-triggered immune activation [29]. In this model, Poly(I: C) stimulation induced a time-dependent increase in DLEU2 expression, accompanied by enhanced IL-6 and IL-8 secretion and increased bronchial epithelial cell apoptosis. IL-6 and IL-8 are key inflammatory mediators in pneumonia. IL-6 drives the acute phase response and promotes lymphocyte activation [30]. IL-8 plays a pivotal role in orchestrating neutrophil-driven airway inflammation and subsequent tissue injury [31]. These findings indicate DLEU2 directly participates in virus-induced epithelial inflammation. Viral infection induces epithelial cell apoptosis, disrupting the alveolar barrier; excessive apoptosis impairs lung tissue repair [32]. Silencing DLEU2 attenuated cytokine production and apoptosis, supporting its pro-inflammatory and pro-apoptotic role in VP.

DLEU2 acts as a ceRNA by sponge-like adsorption of miR-330-5p. miR-330-5p expression is reduced in both severe pneumonia patients and Poly(I: C)-treated epithelial cells. Furthermore, miR-330-5p expression serves as a critical risk factor influencing disease progression. Dual luciferase reporter assays confirmed direct interaction between DLEU2 and miR-330-5p. miR-330-5p exert anti-inflammatory effects in multiple immune-related contexts [17, 33]. Functionally, restoring miR-330-5p expression partially reverses inflammation and apoptosis. These findings support a defensive role for miR-330-5p in VP advancement. Exploring further downstream, we hypothesized that CEBPD is immediate downstream target of miR-330-5p. CEBPD is a transcription factor known to regulate inflammatory gene expression and participate in stress-induced immune responses [34]. CEBPD has been identified as a potential treatment target for acute lung injury [35]. CEBPD is activated in severe COVID-19 patients [36]. Poly(I: C) stimulated CEBPD expression, and luciferase assays confirmed direct binding of miR-330-5p to CEBPD. DLEU2 may uncouple miR-330-5p’s suppression of CEBPD, thereby amplifying inflammatory signaling and promoting epithelial cell apoptosis. This novel DLEU2/miR-330-5p regulatory axis provides new insights into the molecular mechanisms underlying pediatric viral pneumonia and suggests potential avenues for targeted intervention. By integrating clinical observations with mechanistic studies, our findings highlight both the diagnostic and biological significance of DLEU2 in this context.

In terms of clinical translation, DLEU2 shows promise as a minimally invasive biomarker, as it can be detected in plasma-derived exosomes using qRT-PCR, a technique that is widely available in clinical laboratories. The assay requires a relatively small sample volume and can typically be completed within several hours, making it feasible for use in pediatric settings. Blood-based testing is routinely performed in children with suspected viral pneumonia, facilitating integration into existing clinical workflows. However, before clinical implementation, several considerations remain, including standardization of exosome isolation procedures, optimization of detection protocols, and validation in larger, multicenter cohorts. Furthermore, combining DLEU2 with existing clinical indicators or biomarkers may improve diagnostic accuracy and enhance its utility in clinical decision-making.

The limitation should be acknowledged. One limitation of this study is that viral subtype was not included as a covariate in the multivariate analysis. Given the heterogeneity of viral infections and the presence of co-infections in our cohort, differences in pathogen distribution may have influenced disease severity and biomarker expression. Future studies with larger sample sizes and stratified analyses based on viral subtypes are warranted. Poly(I: C) stimulation is a well-established in vitro model, however, it cannot fully recapitulate the complexity of viral infections in vivo. Animal models and primary airway epithelial cells would further strengthen the translational relevance of our findings.

## Conclusion

Elevated DLEU2 may serve as an early indicator of disease progression in children with VP, highlighting its potential utility as a biomarker for screening and severity assessment. Notably, given the observational nature of the clinical data, the association between DLEU2 expression and disease severity does not imply causality. Functional experiments suggest that DLEU2 may contribute to inflammatory responses and epithelial cell apoptosis through the miR-330-5p axis, although further in vivo and clinical validation is required.

## Supplementary Information

Supplementary Material 1.

Supplementary Material 2.

## Data Availability

The datasets used and/or analysed during the current study are available from the corresponding author on reasonable request.
