# Supplementary figures and images for: Exosomal lncRNA DLEU2 aggravates inflammatory injury and apoptosis in pediatric viral pneumonia via the miR-330-5p
Source: Hereditas. 2026 Mar 25;163:57. doi: 10.1186/s41065-026-00665-y (PMC13137524; doi:10.1186/s41065-026-00665-y)

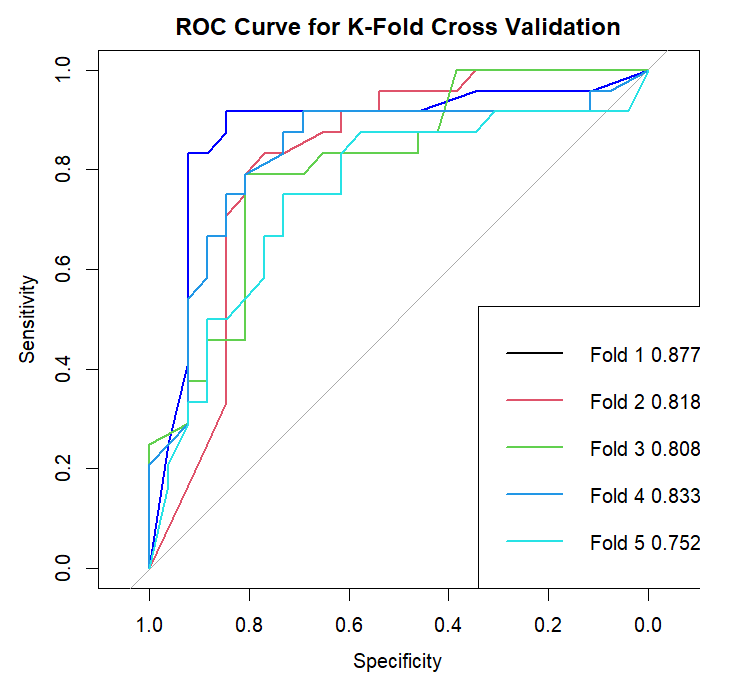

Supplement: Supplementary file 1 — Supplementary Material 1. [file 41065_2026_665_MOESM1_ESM.tiff]
