# Supplementary material for: Exosomal lncRNA DLEU2 aggravates inflammatory injury and apoptosis in pediatric viral pneumonia via the miR-330-5p
Source: Hereditas. 2026 Mar 25;163:57. doi: 10.1186/s41065-026-00665-y (PMC13137524; doi:10.1186/s41065-026-00665-y)
Supplement: Supplementary file 2 — Supplementary Material 2. [file 41065_2026_665_MOESM2_ESM.docx]

**Table S1 Distribution of respiratory viral pathogens in mild and severe pneumonia patients**

| Pathogen composition | Mild Pneumonia (n = 120) | Severe Pneumonia (n = 127) | P-value |
| --- | --- | --- | --- |
| RSV | 36 | 40 | 0.907 |
| HRV | 31 | 6 | < 0.001 |
| ADV | 15 | 30 | 0.035 |
| Flu A/B | 13 | 34 | 0.002 |
| PIV | 12 | 7 | 0.278 |
| HMPV | 5 | 7 | 0.769 |
| HCoV | 4 | 1 | 0.202 |
| HBoV | 2 | 2 | 1.000 |
| CMV | 1 | - | - |
| EBV | 1 | - | - |
| Single-pathogen infection | 99 | 76 | < 0.001 |
| Dual-pathogen infection | 21 | 43 | 0.005 |
| Triple-pathogen infection | - | 8 | - |

Abbreviations: ADV, Adenovirus; CMV, Cytomegalovirus; EBV, Epstein-barr virus; Flu A/B, Influenza virus A/B; HBoV, Human boca virus; HCoV, Human corona virus; HMPV, Human metapneumo virus; HRV, Human rhinovirus; PIV, Parainfluenza virus; RSV, Respiratory syncytial virus;
